# Effectiveness of usual-care cognitive-behavioral therapy for adolescents with depressive disorders rated by parents and patients – an observational study

**DOI:** 10.1186/s12888-021-03404-x

**Published:** 2021-08-24

**Authors:** Daniel Walter, Jana Buschsieweke, Lydia Dachs, Hildegard Goletz, Anja Goertz-Dorten, Claudia Kinnen, Daniela Perri, Christiane Rademacher, Stephanie Schuermann, Paula Viefhaus, Katrin Woitecki, Tanja Wolff Metternich-Kaizman, Elena von Wirth, Manfred Doepfner

**Affiliations:** 1 Department of Child and Adolescent Psychiatry, Psychosomatics and Psychotherapy, Faculty of Medicine and University Hospital Cologne, University of Cologne, Robert-Koch-Str. 10, 50931 Cologne, Germany; 2 School of Child and Adolescent Cognitive Behavior Therapy (AKiP), Faculty of Medicine and University Hospital Cologne, University of Cologne, Pohligstr. 9, 50969 Cologne, Germany

**Keywords:** Routine treatment, Adolescent psychotherapy, Depressive disorder, Cognitive-behavioral therapy, Outpatient clinic

## Abstract

**Background:**

Depressive disorders are common in adolescence and are associated with a wide range of negative long-term outcomes. Highly controlled randomized controlled trials (RCT) provide considerable evidence for the efficacy of cognitive-behavioral therapy (CBT) as a treatment for depression, but studies examining the effectiveness of CBT in clinical settings are very rare .

**Methods:**

In the present observational study, the changes achieved through routine CBT in adolescents with depressive disorders treated in a clinical setting in terms of a university outpatient clinic were analyzed, and compared to a historical control group of adolescents with depressive disorders who had received treatment as usual. At the start and end of treatment (pre- and post-assessment), parent and self-ratings of the German versions of the Youth Self-Report (YSR), the Child Behavior Checklist (CBCL) and rating scales for depressive symptoms (FBB-DES, SBB-DES) were assessed. A total of 331 adolescents aged 11–18 years with complete data were assessed for the main analysis.

**Results:**

The analysis yielded small to large pre-post effect sizes (Cohen’s *d*) for the total sample (*d* = 0.33 to *d* = 0.82) and large effect sizes for adolescents who were rated in the clinical range on each (sub) scale at the start of treatment (*d* = 0.85 to *d* = 1.30). When comparing patients in the clinical range with historical controls, medium to large net effect sizes (*d* = 0.53 to *d* = 2.09) emerged for the total scores in self- and parent rating. However, a substantial proportion of the sample remained in the clinical range at the end of treatment.

**Conclusions:**

These findings suggest that CBT is effective for adolescents with depressive disorders when administered under routine care conditions, but the results must be interpreted with caution due to the lack of a direct control condition.

**Trial registration:**

DRKS, DRKS00021518. Registered 27 April 2020 - Retrospectively registered, http://drks.de

**Supplementary Information:**

The online version contains supplementary material available at 10.1186/s12888-021-03404-x.

## Background

Depressive disorders are common among children and adolescents, with a prevalence between 3 and 12%, an incidence peak around puberty, and a lifetime risk of 15–20% [1, 2]. Affected youth have a greater risk of serious impairments in the areas of psychosocial, family and academic functioning and depressive disorders represent one of the leading causes of disability, morbidity and mortality, considerably increasing the risk of suicide [3, 4]. If adolescent depression is left untreated, there is a considerable risk that the condition will become chronic, and studies have shown that more than two thirds of adolescents with untreated depression relapse within the next 5 years [5]. Depression in adolescence increases the risk of negative long-term outcomes, such as poor physical health, social isolation and poor vocational attainment and achievement (i.e., [6]).

It seems clear that an optimal treatment of this group is essential and unsurprisingly, intensive psychotherapy research examining the treatment of depression has been conducted over the last decades. Such studies have been aggregated into systematic reviews and meta-analyses demonstrating the efficacy of cognitive-behavioral therapy (CBT) (i.e., [7–10]). Moreover, numerous studies have demonstrated the efficacy of antidepressant medication (mainly SSRIs) in the treatment of depressive disorders in children and adolescents (for a summary, see [11]). However, international guidelines consistently recommend CBT and interpersonal therapy (IPT) as the first-line treatment for depression in young people [12, 13].

For CBT, early treatment studies that mostly relied on self-ratings found between-group effect sizes ranging from *d* = .34 [14] to *d* = 1.27 [15], with an average effect size of *d* = .99, indicating a large reduction of depressive symptoms. However, a recent meta-analysis including nearly 450 randomized controlled trials (RCTs) conducted over 50 years of psychotherapy research found a clearly smaller, medium effect size of d = .29 [9]. Moreover, the authors examined the course of these effect sizes over five decades and found that specifically for depression, mean effect sizes had decreased over the assessed period [16]. Only one meta-analysis reported pre-post effect sizes and found a symptom reduction, mainly based on self-ratings, of *d* = 1.23 within the CBT condition and *d* = 0.37 for control conditions (mainly waiting-list or no-treatment), thus suggesting a large symptom reduction during CBT [17].

This strong evidence for CBT found in the above-mentioned meta-analyses was deviated from efficacy trials using randomized controlled study designs. Such RCT designs, which are considered as gold standard within psychotherapy research [18], randomly assign patients to a treatment or a control condition. Patients usually have a specific disorder and meet stringent inclusion and exclusion criteria. Patients in the treatment group receive the specific treatment, which is often delivered by psychotherapists who have been specifically trained for the purpose of the study and are intensively supervised during the treatment. The control group is most often a waiting-list or no-treatment control condition. Many of these studies are performed at university departments and their results can be attributed to the treatment itself due to their high experimental control (high internal validity). However, criticism has been raised regarding the external validity of these studies, as patients, therapists and the treatments themselves often differ considerably from those used in routine care conditions [19]. As such, it has been argued that results from RCTs cannot be generalized to routine care situations [18, 20, 21]. Furthermore, most of the above-mentioned results were obtained by analyzing patients’ self-ratings. It is well known that raters differ considerably regarding the severity of depressive symptoms, and increasing numbers of researchers are therefore calling for the inclusion of multiple informants in order to maximize the objectivity of assessment [9, 22].

Effectiveness studies, on the other hand, examine interventions which are performed under routine care conditions. In these studies, clinically referred patients receive psychotherapy from therapists working in routine care settings and receiving a usual level of supervision [18]. The main advantage of this study design lies in its high external validity: Effectiveness studies can provide evidence that a specific intervention works under real-life conditions. Therefore, both types of studies – efficacy and effectiveness studies – are needed in psychotherapy research. First, an RCT should prove the efficacy of a specific treatment under highly controlled conditions. Second, the effectiveness of the treatment should be demonstrated under routine care conditions. Consequently, a growing number of researchers are calling for effectiveness studies that determine whether the effects found in RCTs can be replicated within routine care settings [18–20, 23].

However, only a very limited number of studies have evaluated the effectiveness of psychotherapy under routine care conditions in real clinical settings. In a systematic review and network meta-analysis, Zhou et al. [10] analyzed 52 RCT studies in which routine therapy served as a control condition. The authors identified a total of *n* = 432 youth with depression who had received all forms of (non-CBT) routine therapy as a control condition (treatment-as-usual). Treatment ingredients and dosages of these control conditions were generally not reported. Compared to a no-treatment condition, in self-rating, a statistically non-significant between-group effect size of *d* = −.18 was found (compared to a waiting-list condition: non-significant, *d* = −.28). A recent systematic review and meta-analysis examined youth with anxiety and/ or depressive symptoms and calculated pre-post changes in effectiveness studies, although the quality of data of the included studies was poor, with important information missing [20]. These studies investigated all forms of treatment as usual, including CBT, psychodynamic therapy, family therapy and pharmacotherapy. Treatments were delivered in different types of outpatient treatment units and the average treatment dosage lay at m = 26.81 treatment sessions. The overall pre-post effect size (Hedges’ g) including different rating perspectives was d = 0.89 for depression, indicating a large reduction of depressive symptoms during treatment. The results of these two papers demonstrated that an unspecified routine therapy, serving as a control condition, produced no treatment effects. In contrast, when delivered in unspecified specialized outpatient treatment units, a mixture of all forms of treatment ingredients may result in large symptom reductions during therapy. However, it is not possible to draw any conclusions about the effectiveness of specific CBT interventions delivered in routine care settings, as although the routine therapy conditions were performed in naturalistic treatment settings, a mixture of different types of therapy and rating perspectives was used. Moreover, due to the limited methodological quality of these effectiveness studies, their results must be interpreted with caution.

A study by our own research group investigated changes under routine CBT within a large sample of *n* = 677 adolescents with all forms of mental disorders in self- and parent rating [24]. Whereas the subgroup of patients with depressive disorders were not analyzed, overall internalizing symptom reductions of *d* = 0.46 in self-rating were found for the whole sample (parent rating: *d* = 0.59).

To the best of our knowledge, only one previous study has investigated the effectiveness of CBT for depression in a routine care setting: Weisz and coworkers [25] randomized *n* = 57 youth aged 8–15 years to a CBT or a treatment-as-usual group. Community clinic therapists were also randomized into two groups. Therapists in the first group received 6 h of training and weekly supervision in CBT, whereas therapists in the usual care (UC) group were asked to apply the treatment procedures that they regularly used in their clinical practice. The mean treatment duration within the CBT group was 16 sessions over 25 weeks. A reduction of depressive symptoms emerged in both groups, with 75% of the patients no longer fulfilling the criteria for a depressive disorder at treatment end. Moreover, no differences were found between the two groups. Pre-post effect sizes were small for self-ratings (*d* = 0.36) and medium-sized for parent ratings (*d* = 0.79). However, the CBT treatment was shorter and therefore less costly.

To summarize, so far, only one study has evaluated CBT for adolescent depression under routine care conditions, and importantly, the interpretation of its results is limited by the fact that CBT was delivered in a manualized form and therapists received only very brief training in CBT. Consequently, no conclusions can be drawn regarding the effectiveness of CBT under routine care conditions in a narrower sense, as it remains unclear whether the manualized CBT delivered within the aforementioned study is comparable to CBT in real clinical settings delivered by regular CBT therapists who have completed in-depth CBT training and have extensive experience in conducting CBT. Furthermore, the relatively small sample size constitutes a further methodological shortcoming of the study by Weisz et al. [25].

We are not aware of any previous studies that systematically investigated the effectiveness of outpatient CBT for the treatment of youth with depression in a real clinical setting. Therefore, the present study aimed to evaluate the effectiveness of CBT in a large sample of clinically referred adolescents with a diagnosis of major depression and applying specific instruments investigating depressive symptoms following ICD-10/ DSM-5. The sample was treated in a university outpatient clinic, representing a naturalistic treatment setting in Germany. Multiple informant perspectives were included and the results were compared to the results from a historical control group having received treatment as usual, which was also used by Weisz and coworkers in the above-mentioned study [25]. We first evaluated changes in depressive symptoms and other mental health problems as rated by patients and their parents from pre-intervention to post-intervention. In addition, we compared the changes in symptom severity to the changes found in the historical control group. This procedure of using a historical control group should help to control for unspecific treatment effects or developmental trends. Nevertheless, due to a lack of randomization, the comparability of the two groups may be reduced and the assumption that the treatment condition in the present study differs from the historical control condition remains unproven [26, 27].

Based on the above-cited results from previous studies, we formulate the following hypotheses:

1. Within the total sample, we expect statistically and clinically significant reductions in self- and parent-rated symptoms of depression and other behavioral and emotional symptoms;
2. Moreover, we expect statistically significant, moderate correlations between the symptom changes rated by patients and parents;
3. When examining a subsample with elevated symptom scores on the respective self- and parent-rating scales assessing depressive symptoms at pre-intervention, we expect statistically and clinically significant reductions, which will be larger in the present study than those in a comparable control group having received treatment as usual, as used in the study by Weisz and coworkers [25];
4. Due to the effects of antidepressant pharmacotherapy, we expect that patients who have received additional antidepressant medication will show statistically larger symptom reductions in self- and parent ratings than those who have not received antidepressant pharmacotherapy.

## Methods

### Participants

Participants were included in this study according to the following criteria:: (1) age 11–18 years, (2) fulfillment of the ICD-10 criteria for any depressive disorder (depressive episode, recurrent depressive disorder, dysthymia, depressive conduct disorder, adjustment disorder with depressed mood, mixed anxiety and depressive disorder), (3) clinically relevant impairment based on clinical judgment, (4) ability to attend treatment appointments once per week, and (5) a positive prognosis for outpatient treatment. The exclusion criteria were severe use of alcohol or other drugs and an indication for inpatient treatment. The study was approved by the ethics committee of the University of Cologne, and all participants and their parents provided written informed consent.

The children and adolescents were either referred to outpatient treatment by their parents, by other inpatient or outpatient departments at the University of Cologne (i.e., Social-Pediatric Center; Department for Child and Adolescent Psychosomatics, Psychiatry and Psychotherapy), or by other clinics and private psychotherapy or psychiatry practices in Cologne and the surrounding area (radius of less than 50 km). They were provided with information on the treatment and the study during a one- to two-hour initial consultation. Moreover, during this consultation, one of the authors assessed the inclusion and exclusion criteria.

From January 2008 to December 2018, a total 597 children and adolescents fulfilled the inclusion criteria and began treatment at the outpatient unit of the University of Cologne. All treatments were completed by December 2018. Patients who received fewer than 10 treatment sessions were excluded, as these patients had only received a brief counseling (*n* = 102; *M* = 5.44 contacts, *SD* = 2.60). A total of 495 patients received at least 10 sessions. For 331 (66.7%) of these patients, pre- and post-test data on self-rating and parent rating were available. This sample was used for the main analyses.

Of the 331 participants, 214 (64.7%) were female. The mean age lay at *M* = 15.87 years (*SD =* 1.69, range from 11.3 to 18.95 years). To measure participants’ intelligence level, we employed the Wechsler Intelligence Scale for Children (WISC [28]), the Kaufman Assessment Battery for Children-2 (K-ABC-II, [29] or the Wechsler Adult Intelligence Scale (WAIS, [30]). Alternatively, the intelligence level was based on clinical rating on the multiaxial classification of child and adolescent psychiatric disorders according to the ICD-10 (ranging from 1 – very high intelligence to 8 – very severe impairment of intelligence [31]). A total of 256 (77.4%) patients had an average intelligence level (*n* = 28 below-average (8.4%), *n* = 47 above-average (14.2%)). At the start of the study, the participants attended the following school types (according to the three-tier German school system): lower track (*n =* 46, 13.9%), medium track (*n =* 73, 22.1%), higher track (*n =* 144, 43.5%), special schools for children with learning disabilities (*n* = 15; 4.5%), special schools for children with emotional and behavioral problems (*n =* 5, 1.5%), vocational schools (*n* = 30, 9.1%). *N* = 18 patients (5.4%) did not attend any type of school. 121 of the patients (36.6%) had repeated a school year at least once, or had gone through an irregular change of school.

Clinical diagnoses were based on a semi-structured clinical interview using DSM- and ICD-based diagnostic checklists [32]. Based on the ICD-10, the patients fulfilled the following clinical diagnoses: depressive episode/ recurrent depressive disorder (*n* = 236, 71.3%), depressive conduct disorder (*n* = 43, 13.0%), adjustment disorder with depressed mood (*n* = 15, 4.5%), mixed anxiety and depressive disorder (*n* = 22, 6.7%), and dysthymia (*n* = 15, 4.5%).

A large proportion of the patients had more than one mental disorder: 127 (38.4%) had two mental disorders and 53 (16.0%) had at least three. The most frequent comorbid mental disorder was anxiety disorders (*n* = 99, 29.9%), followed by ADHD (*n* = 38, 11.5%). 183 (55.3%) of the patients had at least one family member with a mental disorder, and 168 (50.8%) had separated parents. The global functioning at the start of treatment was based on the multiaxial classification of child and adolescent psychiatric disorders according to the ICD-10 [33]. Participants’ global functioning was as follows (ranging from 0 – superior functioning to 8 – persistent inability to maintain minimal personal hygiene/ persistent danger of severely hurting self or others): 1 - satisfactory (*n* = 2, 0.6%), 2 - mild impairment (*n* = 26, 7.9%), 3 - moderate impairment (*n* = 145, 43.8%), 4 - serious impairment in at least one area (*n* = 126, 38.1%), 5 - serious impairment in most of the areas (*n* = 28, 8.5%), 6 - severe and profound impairment in most of the areas (*n* = 4, 1.2%).

### Procedure

Study eligibility was assessed 1–10 weeks before the start of treatment and participants were consecutively included in the study. The first assessment took place within the first five treatment sessions (pre-assessment) and comprised standardized questionnaires completed by patients and parents. The second assessment took place at the end of the treatment (within the last 3 weeks before treatment end) and included ratings by patients and parents (post-assessment).

The present sample overlaps with another sample from our research group displaying heterogeneous mental disorders and investigating changes on internalizing and externalizing problem scales in adolescent and parent rating (*n* = 106; 32.02%) [24].

### Historical controls

The control group for the present study was drawn from the study by Weisz et al. [25]. A sample of 57 children and adolescents aged between 8 and 15 years (*M* = 11.77; *SD* = 2.14) was recruited from seven urban public mental health clinics. Within a project interview, the symptom criteria of minor (MinDD) or major depressive disorder (MDD) or dysthymic disorder (DD) were assessed. The total sample was randomized into a group of *n* = 32 youth who received CBT and another group of *n* = 25 who received treatment as usual (TAU, interventions from multiple theoretical orientations, mostly non-behavioral interventions) – this latter group served as the historical control group in the present study. Study therapists were social workers, doctoral- and Master-level psychologists and other Master-level professionals who were employed in community care clinics where the treatments took place. Therapists had on average 4 years of therapist training and 2 years of additional professional experience prior to the study. Clinical diagnoses of the total sample were as follows: 56.0% MDD, 32.0% MinDD, 12.0% DD. Comorbidity was high, with *M* = 2.76 total diagnoses (*SD* = 1.71) (most frequently oppositional defiant disorder, ADHD, separation anxiety disorder). The mean number of treatment sessions in the TAU condition of the historical control group was *M* = 20.52 (*SD* = 16.07) over nearly 40 weeks. Table 1 shows additional demographic and clinical characteristics of our total sample and the TAU subsample of Weisz and coworkers [25]. The means and standard deviations of our subsamples of youth with elevated symptom scores at the start of treatment on the CBCL Anxious/depressed subscale were *M* = 72.75 (*SD* = 8.05) (CBCL Withdrawn: *M* = 72.19 (*SD* = 8.59); CBCL Externalizing: *M* = 67.43 (*SD* = 5.93)) (see Methods section).

**Table 1:** Sample characteristics of our total sample (*n* = 331) and the total/ TAU sample of Weisz and coworkers [25] (*n* = 25)

	Our total sample	TAU subsample of Weisz and coworkers (2009)
Sex of child (% girls)	64.70	56.0
Mean age in years	15.86	11.5
Mean CBCL Anxious/depressed (T score)	69.20	68.18
Mean CBCL Withdrawn (T score)	69.15	70.59
Mean CBCL Externalizing problems (T Score)	59.47	67.32

### Measures

#### Diagnostic interviews

All clinical diagnoses were based on clinical examinations using the clinical rating scales of the DISYPS [32] as well as a semi-structured clinical interview drawing on the diagnostic criteria of the ICD-10 and DSM-IV. Good internal consistencies (range from α*r* = .69–.95) were found within clinical and field sample. Correlations between clinical ratings based on adolescent and parent interviews lay in the moderate range [32].

#### Parent and self-rating scales

To assess emotional and behavioral problems, the German versions of the parent-rated Child Behavior Checklist (CBCL) and the self-rated Youth Self Report (YSR) [34] were used. The parent form consists of 118 items (self-report: 112 items) which are aggregated into eight narrowband syndrome scales and three broadband scales (Internalizing problems, Externalizing problems, Total problems). Representative German norms are available for parent and self-rating. Reliability and validity of the German versions has been demonstrated [34]. Cronbach’s alpha for the CBCL total scale in our sample was *α*=.95 (YSR: *α* = .92, respectively).

The occurrence of specific depressive symptoms was assessed using the FBB-DES (parent rating) and SBB-DES (self-rating) scales [32]. Both versions consist of 29 symptoms corresponding to the diagnostic criteria for depressive episodes and dysthymic disorder according to ICD-10 and DSM-IV/5. Good reliability and validity of these rating scales have been shown [32]. Cronbach’s alpha for the FBB-DES total scale in our sample was *α*=.89 (SBB-DES: *α* = .91, respectively).

In the historical control group, the Children’s Depression Inventory (CDI) [35] total score was used in self- and parent rating. The CDI consists of 27 items which are rated on a 3-point scale and are scored on five subscales. This widely used instrument has demonstrated good psychometric properties (e.g. [36]).

#### Basic documentation form

The standardized “Basic Documentation Form” [37] records sociodemographic data (i.e. sex, age) as well as treatment characteristics (i.e., treatment duration, number of sessions). It additionally includes the following clinical ratings: (1) global functioning (ranging from 0 = very good functioning in all areas to 8 = needs persistent support 24 h per day) at pre- and at post-assessment based on axis six of the Multiaxial Classification of Child and Adolescent Psychiatric Disorders [38]; (2) the overall clinical improvement (range from 1 = very much improved/ remitted to 5 = worsened) (shortened version of the Clinical Global Impressions Scale-Improvement [39]); and (3) the cooperation of children/adolescents and parents (range from 1 = no cooperation to 5 = very good cooperation).

### Therapy setting and treatment

The study took place in the outpatient clinic of a school of child and adolescent cognitive-behavioral therapy in Germany. The treatments were delivered by postgraduate students with a Master’s degree in psychology or education. The students were in the second half of their training in child and adolescent CBT, which encompasses 5 years and requires 600 sessions of psychotherapy to be delivered during the second half of the training. The psychotherapy sessions during this CBT training are guided by an accredited CBT supervisor (one supervision session every fourth therapy session). The therapies were based on the currently recommended cognitive-behavioral methods for the treatment of depression. Table 2 provides information on the specific treatment modules, as rated by the therapists at the end of treatment in the Basic Documentation Form. Nearly every treatment included patient- and parent-focused interventions and one quarter of the therapies included interventions in schools, delivered for teachers either by telephone or in school. Moreover, one third of all treatments integrated sociotherapy-based interventions. Finally, almost one in four treatments included pharmacotherapeutic interventions, mainly selective serotonin reuptake inhibitors (*n* = 32, 9.7%), other antidepressant medications (*n* = 17, 5.2%) or psychostimulants (*n* = 14, 4.2%). The mean treatment duration was *M* = 17.2 months (*SD* = 8.7), encompassing *M* = 42.89 treatment sessions on average (*SD* = 21.82). All treatment costs were covered by the German health insurance system.

**Table 2:** Most frequent interventions

Intervention	Percentage of the sample (*n* = 331)
Patient-focused interventions in total	99.7
Psychoeducation and cognitive methods	99.1
Token economy	76.4
Social skills training	72.2
Parent−/ family-focused interventions in total	92.7
Psychoeducation and cognitive methods	91.5
Guidance to implement token economy at home	60.7
Methods to enhance the relationship between parents and children/adolescents	52.0
School-focused interventions in total	24.5
Psychoeducation and cognitive methods	22.1
Guidance to implement token economy at school	10.3
Methods to enhance the relationship between teacher and children/adolescents	3.3
Socio-therapeutic interventions in total	33.2
Counseling from social workers	20.2
Social-educational family support	11.5
Medication in total	24.8
SSRI	12.4
Other antidepressants	4.8
Psychostimulants	4.2

### Statistical analysis

The main analyses were conducted for treatments with at least 10 sessions and for which complete data were available for all measures described above (*n* = 331). Two different analyses were conducted to check the representativeness of the sample: First, the sample with fewer than 10 sessions (*n* = 102, brief counseling) was compared to the sample with at least 10 treatment sessions (*n* = 495). Second, the sample with at least 10 treatment sessions and complete data (*n* = 331) was compared to the patients with incomplete data (*n* = 164; excluded due to missing data). Missing data were as follows: CBCL, *n* = 7 at pre-assessment, *n* = 99 at post-assessment, *n* = 23 at both assessments; YSR, *n* = 6 at pre-assessment, *n* = 94 at post-assessment, *n* = 11 at both assessments; FBB-DES, *n* = 9 at pre-assessment, *n* = 107 at post-assessment, *n* = 28 at both assessments; SBB-DES, *n* = 13 at pre-assessment, *n* = 94 at post-assessment, *n* = 15 at both assessments. In a subsequent analysis of representativeness, the missings at pre and post assessment were replaced by multiple imputation for the main scales of the CBCL, YSR, FBB-DES and SBB-DES. All eight variables (CBCL and YSR internal, external and total score, FBB-DES and SBB-DES total score) were imputed together and in total, 20 datasets were created.

Comparisons were conducted with respect to sociodemographic and pre-assessment data in parent and self-rating, as well as clinical ratings of treatment characteristics and effects, using *t*-tests for dependent samples (continuous variables) or Chi-squared tests (dichotomous variables). To determine the magnitude of differences, effect sizes for dependent samples ((M_incomplete_–M_complete_)/SD_pooled_) [40] or odds ratios were calculated.

T-tests for dependent samples were conducted to examine overall changes from pre- to post-assessment for the total sample (*n* = 331) and for the subsamples, and effect sizes for dependent samples were computed to determine the magnitude of changes (*d* = [M_pre_–M_post_]/SD_pre_) [40]. For all analyses, the significance level was set at *α* < 5% and Bonferroni correction was applied (alpha divided by the sum of tests). Furthermore, for the total sample, bivariate correlations of the change within the total scores of the CBCL and YSR as well as the FBB-DES and SBB-DES were computed. While all adolescents had a clinical diagnosis of at least one mental disorder, at the start of treatment, some of the participants rated in the normal range on particular scales according to self- and parent rating. Therefore, further effectiveness analyses included analyses of subsamples of patients with elevated symptom scores at the start of treatment (at least one standard deviation above the mean of the norm group on the analyzed scale at pre-assessment, *T* > 59/ *ST* > 6, clinical range). German norms and cut-offs were used for both the parent and self-rating (T > 59). In order to control for regression to the mean or unspecific treatment effects, the historical control group of Weisz and coworkers [25] was used. As the authors only published means and standard deviations of the T-scores of the usual care (UC) group, these T-scores were used to calculate an effect size (*d*_historical control_) which was subsequently compared to our sample of children/adolescents who rated in the clinical range at pre-assessment using net effect sizes (*d*_therapy group_–*d*_historical control_) [40].

For the assessment of clinical relevance, we combined two criteria [41]: First, we examined whether a participant had changed to normal functioning (*T* < 60), and second, the Reliable Change Index (RCI [41];) was calculated to analyze whether these changes were statistically reliable. These analyses were conducted for the broadband scales of the CBCL and YSR (Externalizing, Internalizing, Total score) as well as the total scores of the FBB-DES and SBB-DES. Patients were divided into the following five groups based on these criteria: (1) improved and clinically normalized; (2) improved but still in a clinical range; (3) unchanged and in a normal range; (4) unchanged and still in a clinical range; (5) worsened. In a final step, we examined whether patients who received monotherapy (CBT) differed from those who additionally received pharmacotherapy on the broadband scales of the CBCL and YSR and on the total scales of the FBB-DES and SBB-DES (ANCOVAs, with post-assessment scores as dependent variables and pre-assessment of the analyzed scale as covariate).

## Results

### Representativeness of complete data

The comparison between participants with complete data who were included in the main analyses (total sample) and participants with incomplete (missing) data who were excluded from the main analyses is presented in Table 3. The following statistically significant differences (all small effect sizes) emerged: Youth with complete data were younger at treatment onset and had a lower score on the YSR Externalizing problems scale. Moreover, therapists rated a better global functioning at treatment onset and a larger improvement during therapy, a larger treatment success for the overall situation, and a better cooperation of youngsters and parents for those with complete data. Finally, the treatment duration was longer.

**Table 3:** Comparison between patients with complete data pre- and post-assessment (n = 331) and those with incomplete data (n = 164)

Variable	Incomplete data *M or % (SD)*	Complete *M or % (SD)*	Test statistic	Statistical significance *P*	Effect size (d) or odds ratio (OR)
Sociodemographic factors					
Age at start of treatment	16.26 (1.65)	15.89 (1.69)	*t* = 2.44	<.05	0.24
Sex (% girls)	57.31	64.65	*χ*^*2*^ = 2.51	n.s.	0.73
Grouped intelligence	3.14 (1.02)	3.00 (0.76)	*t* = 1.69	n.s.	0.15
Relationship status of parents: % separated	56.71	48.04	*χ*^*2*^ *= 3.30*	n.s.	0.71
Parent rating (pre)^a^					
CBCL Internalizing	18.64 (8.89)	19.92 (9.53)	*t* = −1.34	n.s.	0.14
CBCL Externalizing	13.85 (9.82)	12.32 (9.18)	*t* = 1.59	n.s.	0.16
CBCL Total	49.22 (22.67)	48.86 (23.07)	*t* = 0.15	n.s.	0.01
FBB-DES	0.84 (0.48)	0.84 (0.44)	*t = 0.16*	n.s.	0.01
Adolescent rating (pre)^b^					
YSR Internalizing	24.38 (10.96)	23.29 (11.16)	*t* = 0.98	n.s.	0.10
YSR Externalizing	15.18 (8.49)	13.30 (7.53)	*t* = 2.42	<.05	0.23
YSR Total	60.76 (23.40)	57.58 (23.97)	*t* = 1.35	n.s.	0.13
SBB-DESSBB-DES	1.10 (0.49)	1.02 (0.53)	*t = 1.60*	n.s.	0.17
Therapist rating					
Global functioning (pre)	3.72 (0.84)	3.50 (0.84)	*t* = 2.75	<.01	0.26
Improvement global functioning (pre-post)	0.87 (1.32)	1.42 (1.31)	*t =* −4.38	<.001	0.41
Treatment success for overall situation (post)	3.74 (1.46)	3.37 (1.15)	*t* = 3.07	<.05	0.27
Cooperation of youngster (post)	3.42 (1.14)	3.89 (0.94)	*t* = −4.89	<.001	0.43
Cooperation of parent (post)	3.75 (2.20)	4.18 (1.74)	*t* = −2.38	<.05	0.21
Treatment duration (years)	1.18 (0.71)	1.43 (0.73)	*t* = −3.66	<.001	0.35

^a^Parent rating (raw scores): complete data of *n* = 331 cases were compared to *n* = 134 incomplete cases having pre-assessment data

^b^Adolescent rating (raw scores): complete data of *n* = 331 cases were compared to *n* = 147 incomplete cases having pre-assessment data

The comparison between patients with fewer than 10 appointments and the rest of the sample revealed no statistically significant differences between the two groups for most of the variables. Differences were found for four variables: The group with a shorter treatment duration was more likely to have separated parents (*OR* = 1.93), and had a lower therapist-rated global functioning at treatment onset (small effect, *d* = 0.22). Moreover, they showed less improvement during treatment (large effect, *d* = 1.09) and therapists rated the treatment success for the overall situation to be inferior (medium effect, *d* = 0.66; see Supplementary Table 1, available online).

### Treatment effectiveness

When analyzing the total sample (*n* = 331), highly significant symptom reductions emerged on all broadband scales of the CBCL and YSR as well as the total scales of the FBB-DES and SBB-DES from pre- to post-assessment, with small to large effect sizes ranging from *d* = 0.33 to *d* = 0.82 (see Table 4). The inclusion of the imputed data resulted if at all to only very small effect size reductions of between Δd = 0.00 and Δd = 0.07.

**Table 4:** Changes in behavioral and emotional problems from pre- to post-assessment on the broadband scales of the CBCL, YSR, and the total scales of the FBB-DES and SBB-DES for the total sample (raw scores, *n* = 331)

	Parent rating (CBCL, FBB-DES)					Adolescent rating (YSR, SBB-DES)						
	Pre-assessment		Post-assessment		*t*-test		Pre-assessment		Post-assessment		*t*-test	
	*M*	*SD*	*M*	*SD*	*t*	d^*^	*M*	*SD*	*M*	*SD*	*t*	d*
CBCL/ YSR												
Internalizing problems	19.92	9.53	12.32	8.94	14.56	0.79	23.29	11.16	14.68	10.58	15.50	0.77
Externalizing problems	12.33	9.18	8.43	7.54	4.70	0.42	13.30	7.53	10.82	6.95	7.19	0.33
Total problems	48.86	23.07	31.69	21.86	19.50	0.74	57.58	23.97	39.95	23.24	14.78	0.74
FBB-DES/ SBB-DES	0.84	0.44	0.48	0.43	13.08	0.81	1.02	0.53	0.59	0.47	15.42	0.82

*All *p* < .001

A moderate, statistically highly significant correlation (*r* = .40, *p* < .001) was found between the symptom changes on the total scores of the CBCL and YSR (FBB-DES and SBB-DES: moderate correlation: *r* = .44, *p* < .001, respectively).

Mean comparisons of pre- and post-assessment on all scales of the CBCL, YSR, FBB-DES and SBB-DES for patients rated in the clinical range (T > 59; ST > 6) on each scale at the start of treatment yielded highly significant symptom reductions, with large effect sizes in parent and self-ratings (range: *d* = 0.85 to *d* = 1.30; see Supplementary Table 2, available online).

### Comparison with historical controls

Table 5 presents the T-score and stanine means, standard deviations and effect sizes of the CBCL and YSR broadband scales as well as the instruments assessing depressive symptoms (FBB-DES, SBB-DES, CDI-P, CDI) for patients rated in the clinical range at the start of treatment and for the historical control group. For the group of treated patients, large effect sizes (ranging from *d* = 1.13 to *d* = 2.43) were found for the change in symptoms from pre- to post-assessment, whereas the historical control group mostly showed small to medium symptom reductions (CDI-P total score: large effect size; range *d* = 0.34 to *d* = 1.20). When comparing the effect sizes of these two groups, the net effect sizes were medium to large, in favor of the treatment group (ranging from *d* = 0.53 to *d* = 2.09).

**Table 5:** T-Score and stanine means, standard deviations and effect sizes for the subsample of patients rated in the clinical range at pre-assessment (CBCL, FBB-DES, SBB-DES) and for the historical control group (CBCL, CDI-P, CDI) that received treatment as usual [25]

		Patients rated in the clinical range					Historical controls						
		*N* (%) of total sample	Pre-assessment		Post-assessment		Pre-assessment			Post-assessment			
			*M*	*SD*	*M*	*SD*	*d*	*M*	*SD*	*M*	SD	*d*	*d*_*net*_^*a*^
CBCL	Anxious/ depressed	266 (80.4)	72.75	8.05	63.15	9.83	1.19	68.18	10.33	62.54	6.21	0.66	0.53
	Withdrawn	274 (82.8)	72.19	8.59	62.24	9.65	1.16	70.59	7.08	64.77	13.65	0.54	0.61
	Externalizing	165 (49.8)	67.44	5.93	60.72	8.45	1.13	67.32	9.38	60.85	12.33	0.59	0.54
FBB-DES/ CDI-P^b^	Total score	278 (83.4)	8.40	0.78	6.64	2.10	2.26	18.56	5.92	11.19	6.32	1.20	1.06
SBB-DES/ CDI^b^	Total score	264 (79.8)	8.40	0.77	6.53	1.86	2.43	11.29	7.93	8.47	8.44	0.34	2.09

^a^net effect size: *d*_therapy group_–*d*_historical control_

^b^raw scores

### Clinical significance

Results regarding the clinical significance of the changes on the CBCL and YSR broadband scales and the FBB-DES/ SBB-DES for the total sample and for the subsample rated in the clinical range at pre-assessment on each scale are presented in Table 6.

**Table 6:** Clinical significance of changes in parent and adolescent ratings on the broadband scales of CBCL and YSR and the FBB-DES and SBB-DES total scale

				Worsened	Unchanged and still in a clinical range	Unchanged and in a normal range	Improved and still in a clinical range	Improved and clinically normalized
			*N*	*n* (%)	*n* (%)	*n* (%)	*n* (%)	*n* (%)
Parent rating								
CBCL	Total sample	Internalizing Problems	331	13 (3.9)	132 (39.9)	60 (18.1)	47 (14.2)	79 (23.9)
		Externalizing Problems	331	13 (3.9)	66 (19.9)	160 (48.3)	23 (6.9)	69 (20.8)
		Total Problems	331	18 (5.4)	80 (24.2)	63 (19.0)	66 (19.9)	104 (31.4)
	Rated in a clinical range at pre-assessment	Internalizing Problems	286	8 (2.8)	116 (40.6)	26 (9.1)	53 (18.5)	83 (29.0)
		Externalizing Problems	165	7 (4.2)	54 (32.7)	16 (9.7)	28 (17.0)	60 (36.4)
		Total Problems	263	11 (4.2)	70 (26.6)	11 (4.2)	71 (27.0)	100 (38.0)
FBB-DES	Total sample	Total score	331	26 (7.9)	81 (24.5	63 (19.0)	71 (21.5)	90 (27.2)
	Rated in a clinical range at pre-assessment	Total score	278	22 (7.9)	69 (24.8)	25 (9.0)	68 (24.5)	94 (33.8)
Adolescent rating								
YSR	Total sample	Internalizing Problems	331	9 (2.7)	107 (32.3)	68 (20.5)	41 (12.4)	106 (32.0)
		Externalizing Problems	331	18 (5.4)	56 (16.9)	183 (55.3)	7 (2.1)	67 (20.2)
		Total Problems	331	16 (4.8)	76 (23.0)	60 (18.1)	44 (13.3)	135 (40.8)
	Rated in a clinical range at pre-assessment	Internalizing Problems	267	8 (3.0)	84 (31.5)	15 (5.6)	51 (19.1)	109 (40.8)
		Externalizing Problems	104	2 (1.9)	37 (35.6)	4 (3.8)	11 (10.6)	50 (48.1)
		Total Problems	231	8 (3.5)	57 (24.7)	11 (4.8)	51 (22.1)	104 (45.0)
SBB-DES	Total sample	Total score	331	9 /2.7)	96 (29.0)	87 (26.3)	50 (15.1)	89 (26.9)
	Rated in a clinical range at pre-assessment	Total score	264	11 (4.2)	65 (24.6)	24 (9.1)	63 (23.9)	101 (38.3)

A clinically significant deterioration at the end of treatment was only found in a relatively small percentage of the total sample (2.7 to 7.9%). In the subsample rated in the clinical range at the start of treatment, the deterioration rates lay between 1.9 and 7.9% across the (sub)scales. Between 29.0 and 48.1% of these participants were improved and clinically normalized at the end of treatment, and a further 3.8 to 9.7% were in the clinically normal range and did not show a clinically significant change. With regard to the parent-rated CBCL total score, 42.2% of the subsample in the clinical range at the start of treatment were in the normal range at the end of treatment, while 57.8% remained in the clinical range with a symptom level of T ≥ 60 (FBB-DES total score: 42.8% normalized, 57.2% clinical range (ST ≥ 7) at treatment end). With regard to the adolescent-rated YSR total score, 49.8% of the subsample lay in the normal range and 50.2% remained in the clinical range at the end of treatment (SBB-DES total score: 47.4% normalized, 52.6% in the clinical range at treatment end). Moreover, with regard to the CBCL and YSR total score, 65.0 and 67.1% of the subsample, respectively, showed clinically significant improvements in parent and self-ratings (58.3% (FBB-DES) and 62.2% (SBB-DES)).

### Effects of additional antidepressant pharmacotherapy

Comparisons between the group of patients who received CBT alone and the group additionally receiving antidepressant psychopharmacotherapy (*n* = 57; 17.2% of the total sample) revealed the following statistically significant differences: Patients who received CBT and antidepressant medication were older at the start of treatment (*t* = − 3.020; *p* < .01; *d* = 0.48) and showed more severe symptoms of mental disorders at the post-assessment on the following scales: CBCL Internalizing problems (*t* = − 2.673; *p* < .01; *d* = 0.38), YSR Internalizing problems (*t* = − 4.164; *p* < .001; *d* = 0.62), YSR Total problems (*t* = − 2.302; *p* < .05; *d* = 0.34), SBB-DES (*t* = − 3.397; *p* < .01; *d* = 0.50).

Nevertheless, when pre-treatment scores were entered as covariates (ANCOVA), only two out of eight scales differed significantly between the two groups at the post-treatment assessment (YSR Internalizing problems: *F* = 3.93, *df* = 1, *p* < .05, eta^2^ = .01; SBB-DES: *F* = 7.05, *df* = 1, *p* < .01, eta^2^ = .02) – patients having received an antidepressant medication rated themselves as having more internalizing / depressive symptoms at the end of treatment.

## Discussion

In the present effectiveness study, we investigated the course of parent- and adolescent-rated behavioral and emotional symptoms of mental disorders in a sample of clinically referred adolescents with depressive disorders undergoing routine outpatient CBT in a clinical setting. The treatment was delivered by psychologists and educationalists with advanced training in CBT in an outpatient clinic. Changes were analyzed for different subsamples and for the total sample, and were compared to a historical control group of patients with depression who had received treatment as usual. Furthermore, the clinical relevance of these changes was assessed and improvements during treatment were compared between patients who received CBT and patients who received CBT plus antidepressant psychopharmacotherapy.

The results revealed statistically highly significant reductions of depressive symptoms and symptoms of other mental disorders in the total sample, as rated by patients and their parents. Mostly large symptom reductions emerged in the subsample which displayed elevated symptom scores on the respective analyzed scales at the start of treatment. Additionally, correlations between parent- and adolescent-rated symptom reductions as well as between parent and adolescent ratings for a broad range of behavioral and emotional problems were moderate and statistically highly significant. This suggests that in terms of their ratings concerning symptom reductions during treatment, adolescents and their parents show moderate levels of agreement with one another.

Our analyses on clinical significance show that a larger share of the sample was clinically normalized at the end of treatment, but more than half of the sample remained in a clinical range. In sum, our hypotheses 1 and 2 can be mostly confirmed, as many youngsters showed a statistically and clinically significant reduction of mental disorder symptoms during treatment. Future analyses of differential effects should be conducted to determine which patients benefit from the delivered treatment, and how the treatment may be improved in order to reduce the proportion of adolescents who are rated in a clinical range by either informant (parents, self) at the end of treatment.

As the present effectiveness study did not use a randomized controlled design, a historical control group was used to control for regression to the mean and unspecific treatment effects. The study by Weisz and coworkers [25], which investigated therapies of youth with depression, is of interest in this regard, as patients and therapists were recruited within routine care settings. Nevertheless, it is important to take into account some relevant differences between our sample and the historical control sample when interpreting the present findings: The historical control group was (1) on average about 4 years younger, (2) less clinically impaired at the start of treatment (nearly half of the sample with MinDD or DD compared to 12.2% of our sample), (3) differed in terms of in- and exclusion criteria and had a much shorter treatment than the routine CBT treatment investigated in our study (average: 20 treatment sessions in 40 weeks vs. 44 treatment sessions in 73 weeks) and (4) a substantially smaller sample size. Due to these differences, we did not conduct direct comparisons in terms of testing for statistically significant differences between the two samples. When comparing this historical control group which received treatment as usual with a subsample of our participants with similar T-scores on the CBCL scales at intake (patients rated in the clinical range at start of treatment), medium to large net effect sizes in favor of our routine CBT group emerged in self- and parent rating, thus confirming our third hypothesis. One possible explanation for the superiority of our routine CBT treatment compared to the non-superiority of the CBT condition within the study by Weisz and coworkers [25] is that compared to their brief CBT training, the therapists who provided our routine therapies had much more CBT knowledge and experience, with two to 5 years of CBT training. On the other hand, it has to be kept in mind that the treatment length and intensity in our study was much higher in terms of substantially more treatment sessions. Therefore, it cannot be ruled out that the higher effects may be attributable to the different treatment intensity. Future studies on differential effects will have to investigate the potential influence of variables such as treatment intensity/ duration or the level of CBT training on symptom reductions. Despite the limitations in terms of the comparability of these two groups, through the use of a historical control group to monitor regression and unspecific effects, we can assume that the symptom reductions found in the present study are not solely attributable to developmental trends or regression effects.

It is difficult to draw comparisons between the results of our observational study and previous published studies which included routine therapy, as study designs, treatments, therapists and sample characteristics differed. Nevertheless, it seems important to review their findings. Three reviews/ meta-analyses are of special interest in this regard. First, the meta-analysis by Michael and Crowley [17] examined 15 controlled studies and reported a small pre-post effect size of *d* = .37 in self-rating for any form of control condition (mainly waiting-list or no-treatment). Second, the network meta-analysis by Zhou and coworkers [10] analyzed 52 studies and found no treatment effect at all in self-rating − all forms of routine treatment were as effective as a waiting-list or no-treatment condition. The pre-post effect sizes found in our total sample were large (*d* = .82 in self-rating and *d* = .81 in parent rating; and in subsamples with elevated symptom scores at the start of treatment on the respective analyzed scale: *d* = 1.20 in self-rating and *d* = 1.05 in parent rating), indicating a statistically significant, medium to large reduction of depressive symptoms during routine CBT. When relating these findings to one another, it can be concluded that routine CBT is more effective than waiting-list, no-treatment or other forms of routine psychotherapy if used as control conditions in RCT studies. However, in a recent review and meta-analysis, Bear and coworkers [20] investigated different forms of routine therapy that were delivered in unspecified specialized outpatient treatment units with more than half of the studies using a non-controlled pre-post design. Overall, the authors found a large reduction of depressive symptoms across all forms of routine therapy and across different raters (d = 0.89). This result is comparable to the pre-post effect sizes found in our total sample. Moreover, in self-rating, a recovery rate of 40% was found, which, depending on the respective instrument, is nearly equivalent to the recovery rates in our sample (YSR Total problems: 45.0%; SBB-DES total score: 38.3%). Therefore, our results are in line with the limited previous research on all forms of routine therapy, and add important knowledge to the research field by identifying large symptom reductions during CBT interventions delivered in a routine care setting. In this respect, it is important to note that especially in terms of depression, unspecific treatment effects based on patients’ expectations are particularly high. As such, specific methods potentially explain only a smaller proportion of the total symptom reduction (see for instance [42]. When relating our results to the interesting work of Bear and coworkers [20], it has to be kept in mind that the studies included in their meta-analysis were mostly of poor methodological quality, for instance key information was missing, and some studies did not assess clinical diagnoses using structured clinical interviews or had limited sample sizes. Moreover, the outcome scores of all included studies were aggregated into one single score. Finally, when taking into account our subsamples of patients with elevated symptom scores on the respective scale at the start of treatment, larger effect sizes were found, which exceed those found in the aforementioned meta-analysis. This might indicate a potential superiority of routine CBT compared to other forms of routine therapy. Future studies using an RCT design and including an active control condition should focus on variables that might influence treatment effectiveness, such as treatment components, dosage, rater effects or sample characteristics.

Compared to earlier, highly controlled efficacy studies reporting pre-post effect sizes of d = 1.23 [17] and between-group effect sizes (mostly based on self-ratings) of d = .34 to d = 1.27 [14, 15], our effect sizes within the total sample are clearly smaller. However, the overall effect size found in the recent meta-analysis by Weisz and coworkers [9] was clearly smaller (d = .29). Nevertheless, the difference between these highly controlled efficacy studies and our results is presumably due to the fact that the efficacy studies differed considerably from our effectiveness study in terms of patients and treatment characteristics. The efficacy studies mostly recruited samples specifically for the purpose of the respective studies, using very strict inclusion and exclusion criteria. Moreover, the therapists were intensively trained for the studies and received a large amount of supervision. By contrast, our sample was very heterogeneous with regard to symptoms and comorbid disorders, and comprised clinically referred young people with serious clinical impairments. As it is well known that comorbidity may negatively affect treatment outcome (e.g., [23]), this heterogeneity constitutes a major challenge within effectiveness studies. Although every patient in our study had a depressive disorder, depending on the rating scale, only 44.7 to 82.8% of the total sample lay in the clinical range at the start of treatment on scales assessing depressive symptoms in parent rating, and between 26.3 and 76.4% in adolescent rating, which might be attributed to factors such as dissimulation. For this reason, the scope for symptom reduction during treatment is reduced on these scales. In this context, our analysis of the subsample with elevated symptom scores on the analyzed scale at the start of treatment is of special interest: Large effect sizes were found, which are comparable to the pre-post effect sizes reported for the RCTs assessing manualized CBT that mostly relied on self-rating [17]. Our study therefore adds important knowledge to the research field by demonstrating patient-rated symptom reductions under CBT delivered under routine care conditions that are comparable to highly controlled studies. Due to considerable differences between raters, researchers are increasingly calling for the inclusion of multiple informants in order to optimize assessment objectivity (e.g., [9, 22]). Nevertheless, most of the published studies relied on self-ratings. One particular strength of our study is that besides the assessment of self-ratings, we also investigated parent-rated symptom changes, and our results show that the reported reductions of depressive symptoms were high but slightly inferior in parent rating compared to self-rating (*d* = 1.05 vs. *d* = 1.20). Possibly, these findings may be attributed to the fact that several depressive symptoms relating to depressive thoughts and feelings can be described more accurately by the patients themselves than by their parents.

Our comparisons of patients who received CBT alone with patients who additionally received antidepressant medication revealed no group differences for most variables. However, adolescents with additional antidepressant medication reported more internalizing problems and more depressive symptoms at the end of treatment (small effect). This suggests that the main effects found in our total sample might not be attributed to the effects of the pharmacological interventions. However, we cannot confirm our hypothesis that stronger treatment effects can be found in patients with additional antidepressant medication. Although for the most part, there were no differences between these two groups at the start of treatment, one possible interpretation is that patients with an indication for additional pharmacotherapy may need this combined treatment in order to attain treatment effects that are comparable to the effects of CBT in patients without an indication for additional pharmacotherapy.

Our study comprised an average treatment duration of 17 months, and an intensity of almost 43 sessions, thus clearly exceeding previous RCTs and naturalistic studies. Therefore, future studies should be conducted to clarify whether less extensive routine CBT interventions are similarly effective.

The lack of a control condition for the total sample constitutes the most important limitation of our observational study. As such, we are unable to rule out whether the observed changes may be caused by confounding factors other than the treatment, for instance natural developmental trends. However, several studies have demonstrated the stability of mental disorders in adolescents over one to 3 years. For example, a representative cross-sectional study comprising nearly 3000 4–18-year-olds in Germany found no significant decreases in behavioral and emotional problems (assessed using the CBCL and YSR) with increasing age over a period of two to 3 years [43].

Although the therapists (in training) in the present study were guided by supervisors in terms of implementing the CBT, and had regular discussions about the treatment sessions, we did not formally assess treatment integrity. Furthermore, while the therapies were performed in a routine care setting in terms of a university outpatient clinic, and were delivered by therapists with advanced CBT training, future studies should examine whether this type of therapy differs from therapy delivered by therapists in outpatient units or private practice under routine care conditions. A further limitation pertains to the representativeness of the analyzed data: Only patients with at least 10 treatment sessions were included in the analysis, and due to missing data, it was not possible to include every treatment in the analysis. When comparing patients included in the analysis with those who had incomplete data, we found that the included patients were significantly less impaired at the start of treatment, and that therapists rated the treatments as more effective, although the differences between groups were small. In terms of the comparison between patients with fewer than 10 appointments (brief counseling) and those with longer treatments, we found that the two groups were comparable with respect to sociodemographic factors and clinical impairment. However, therapists rated the group of excluded patients with brief counseling to be less cooperative, to show a minor treatment success for the overall situation, and to have less improvement in global functioning in terms of treatment dropouts. Therefore, it cannot be ruled out that our results overestimate the effectiveness of routine CBT in youth with depressive disorders, even though after imputing missing data, if at all only very small reductions of effect sizes were found.

Future studies should thus examine the most common reasons for treatment dropout in order to analyze how treatment dropouts could be reduced. Additional specific instruments and additional raters, such as teachers, should be included. To reduce missing data, especially at treatment end, a sufficient data monitoring should be included in the study process. Moreover, to assess the stability of the changes observed during treatment, follow-up assessments are needed. Finally, future studies should try to integrate a direct comparison group, e.g. in the form of a treatment-as-usual or a waiting-list control condition.

## Conclusion

The present effectiveness study on routine CBT of adolescents with depressive disorders is the first to demonstrate the potential benefits of this kind of treatment for adolescents within a natural, clinical treatment setting while investigating a large sample of clinically referred adolescents. As such, our findings support the results of RCTs demonstrating the efficacy of CBT for children and adolescents in more controlled but less representative conditions.

## Supplementary Information

**Additional file 1: Supplementary Table 1.** Comparison of patients with fewer than 10 appointments (*n* = 102, brief counseling) with those who had at least 10 treatment sessions (*n* = 495, longer treatment). **Supplementary Table 2.** Changes in behavioral and emotional problems from pre- to post-assessment on the scales of the CBCL, YSR, FBB-DES and SBB-DES for the subsample of patients rated in the clinical range at the start of treatment.

## Data Availability

The datasets used and/or analyzed during the current study are available from the corresponding author on reasonable request.

## Abbreviations

CBT: Cognitive-behavioral therapy
CBCL: Child Behavior Checklist
CDI: Children’s Depression Inventory
DISYPS: Diagnostik-System für psychische Störungen nach ICD-10 und DSM-V für Kinder und Jugendliche [DISYPS: Diagnostic system for psychiatric disorders in children and adolescents]
FBB-DES: Fremdbeurteilungsbogen für depressive Störungen [parent rating for depressive disorders]
IPT: Interpersonal therapy
K-ABC-II: Kaufman Assessment Battery for Children-2
RCT: Randomized controlled trials
RCI: Reliable change index
SBB-DES: Selbstbeurteilungsbogen für depressive Störungen [self-rating for depressive disorders]
SSRI: Selective serotonin reuptake inhibitors
TAU: Treatment as usual
WISC: Wechsler Intelligence Scale for Children
WAIS: Wechsler Adult Intelligence Scale
YSR: Youth Self-Report
