# Supplementary material for: Effectiveness of usual-care cognitive-behavioral therapy for adolescents with depressive disorders rated by parents and patients – an observational study
Source: BMC Psychiatry. 2021 Aug 24;21:423. doi: 10.1186/s12888-021-03404-x (PMC8386089; doi:10.1186/s12888-021-03404-x)
Supplement: Supplementary file 1 — Additional file 1: Supplementary Table 1. Comparison of patients with fewer than 10 appointments (n = 102, brief counseling) with those who had at least 10 treatment sessions (n = 495, longer treatment). Supplementary Table 2. Changes in behavioral and emotional problems from pre- to post-assessment on the scales of the CBCL, YSR, FBB-DES and SBB-DES for the subsample of patients rated in the clinical range at the start of treatment. [file 12888_2021_3404_MOESM1_ESM.docx]

**Supplementary Table 1**. Comparison of patients with fewer than 10 appointments (*n* = 102, brief counseling) with those who had at least 10 treatment sessions (*n* = 495, longer treatment)

|  | Variable | Brief counseling | | Longer treatments | | Test statistic^*^ | Statistical significance  *p* | Effect size (*d*) or odds ratio (*OR*) |
| --- | --- | --- | --- | --- | --- | --- | --- | --- |
|  |  | *M* or  % | *SD* | *M* or % | *SD* |  |  |  |
| Sociodemographic factors | |  |  |  |  |  |  |  |
|  | Age at start of treatment | 16.17 | 1.50 | 16.00 | 1.69 | *t* = 0.98 | n.s. | *d* = 0.11 |
|  | Sex: % girls | 58.82 | | 62.22 | | *chi^2^* = 0.41 | n.s. | *OR* = 0.8 |
|  | Grouped intelligence | 3.02 | 0.38 | 2.98 | 0.55 | *t* = 0.78 | n.s. | *d* = 0.09 |
|  | Relationship status of parents: % separated | 66.67 | | 50.91 | | *chi^2^* = 8.44 | <.01 | *OR* = 1.93 |
| Parent rating (pre)^1^ | |  |  |  |  |  |  |  |
|  | CBCL Internalizing | 18.00 | 9.16 | 19.55 | 9.36 | *t* = -1.25 | n.s. | *d* = 0.56 |
|  | CBCL Externalizing | 15.27 | 10.37 | 12.77 | 9.39 | *t* = 1.98 | n.s. | *d* = 0.12 |
|  | CBCL Total | 49.14 | 23.41 | 48.96 | 22.93 | *t* = 0.06 | n.s. | *d* = 0.01 |
|  | FBB-DES | 0.86 | 0.52 | 0.84 | 0.45 | *t* = 0.35 | n.s. | *d* = 0.05 |
| Adolescent rating (pre)^2^ | |  |  |  |  |  |  |  |
|  | YSR Internalizing | 23.01 | 10.94 | 23.62 | 11.10 | *t* = -0.42 | n.s. | *d* = 0.05 |
|  | YSR Externalizing | 14.51 | 8.43 | 13.89 | 7.88 | *t* = 0.61 | n.s. | *d* = 0.08 |
|  | YSR Total | 57.74 | 24.76 | 58.56 | 23.81 | *t* = -0.26 | n.s. | *d* = 0.03 |
|  | SBB-DES | 1.13 | 0.51 | 1.04 | 0.52 | *t* = 1.22 | n.s. | *d* = 0.16 |
| Therapist rating | |  |  |  |  |  |  |  |
|  | Global functioning (pre) | 3.77 | 1.31 | 3.57 | 0.85 | *t* = 1.98 | <.05 | *d* = 0.22 |
|  | Improvement global functioning (pre-post) | -0.19 | 1.16 | 1.24 | 1.33 | *t* = -10.03 | < .001 | *d* = 1.09 |
|  | Treatment success for overall situation (post) | 4.65 | 3.15 | 3.50 | 1.27 | *t* = 6.09 | < .01 | *d* = 0.66 |
|  | Cooperation of youngster (post) | 3.52 | 2.51 | 3.74 | 1.03 | *t* = -1.42 | n.s. | *d* = 0.15 |
|  | Cooperation of parents (post) | 4.26 | 2.85 | 4.04 | 1.91 | *t* = 0.98 | n.s. | *d* = 0.11 |

^1^ Parent rating: complete data of *n* = 465 cases were compared to *n* = 102 cases of brief counseling having pre-assessment data

^2^ Adolescent rating: complete data of *n* = 477 cases were compared to *n* = 67 cases of brief counseling having pre-assessment data

**Supplementary table 2**. Changes in behavioral and emotional problems from pre- to post-assessment on the scales of the CBCL, YSR, FBB-DES and SBB-DES for the subsample of patients rated in the clinical range at the start of treatment

|  |  | Parent rating | | | | | | | Adolescent rating | | | | | | |
| --- | --- | --- | --- | --- | --- | --- | --- | --- | --- | --- | --- | --- | --- | --- | --- |
|  |  | *N* (%) of total sample | Pre-assessment | | Post-assessment | | *t*-test | | *N* (%) of total sample | Pre-assessment | | Post-assessment | | *t*-test | |
|  |  |  | *M* | *SD* | *M* | *SD* | *t* | *d*^*^ |  | *M* | *SD* | *M* | SD | *t* | *d** |
| CBCL/ YSR | Social Withdrawal | 274 (82.8) | 8.20 | 3.17 | 4.74 | 3.50 | 16.49 | 1.09 | 249 (75.2) | 7.90 | 2.62 | 4.73 | 3.06 | 15.64 | 1.21 |
|  | Somatic Complaints | 236 (71.3) | 5.40 | 2.76 | 3.17 | 2.94 | 10.88 | 0.81 | 213 (64.4) | 6.48 | 2.61 | 3.74 | 3.13 | 12.24 | 1.05 |
|  | Anxiety/ Depression | 266 (80.4) | 11.63 | 4.70 | 6.65 | 4.91 | 16.80 | 1.06 | 250 (75.5) | 16.55 | 6.21 | 9.76 | 6.72 | 15.10 | 1.09 |
|  | Social Problems | 160 (48.3) | 5.06 | 2,36 | 3.07 | 2.62 | 9.99 | 0.84 | 148 (44.7) | 6.30 | 2.14 | 3.62 | 2.66 | 12.91 | 1.25 |
|  | Thought Problems | 177 (53.5) | 3.17 | 1.66 | 1.43 | 1.77 | 12.63 | 1.05 | 213 (64.4) | 3.90 | 2.17 | 2.05 | 2.22 | 10.76 | 0.85 |
|  | Attention Problems | 200 (60.4) | 8.38 | 3.25 | 5.71 | 4.66 | 8.32 | 0.82 | 198 (59.8) | 9.68 | 2.23 | 6.77 | 3.22 | 12.21 | 1.30 |
|  | Rule-breaking Behavior | 161 (48.6) | 5.87 | 3.01 | 3.75 | 3.07 | 7.52 | 0.70 | 121 (36.5) | 7.69 | 2.19 | 5.47 | 3.30 | 7.12 | 1.01 |
|  | Aggressive Behavior | 150 (45.3) | 14.77 | 5.54 | 9.50 | 5.79 | 9.92 | 0.95 | 108 (32.6) | 15.03 | 3.80 | 10.83 | 4.87 | 9.08 | 1.11 |
|  | Internalizing Problems | 286 (86.4) | 22.05 | 8.43 | 13.01 | 8.92 | 17.09 | 1.07 | 267 (80.7) | 26.89 | 9.04 | 16.57 | 10.51 | 16.59 | 1.14 |
|  | Externalizing Problems | 165 (49.8) | 19.26 | 7.97 | 12.80 | 7.79 | 9.20 | 0.81 | 104 (31.4) | 22.42 | 5.11 | 15.89 | 7.05 | 9.52 | 1.28 |
|  | Total Problems | 263 (79.5) | 56.49 | 19.39 | 35.46 | 21.82 | 16.01 | 1.08 | 231 (69.8) | 68.69 | 19.26 | 46.10 | 23.10 | 15.54 | 1.17 |
| FBB-DES/ SBB-DES | Total score | 278 (83.4) | 0.93 | 0.40 | 0.51 | 0.44 | 13.99 | 1.05 | 264 (79.8) | 1.19 | 0.44 | 0.66 | 0.48 | 17.30 | 1.20 |

* all: *p* < .001
